# Nanocone-Array-Based Platinum-Iridium Oxide Neural Microelectrodes: Structure, Electrochemistry, Durability and Biocompatibility Study

**DOI:** 10.3390/nano12193445

**Published:** 2022-10-01

**Authors:** Qi Zeng, Shoujun Yu, Zihui Fan, Yubin Huang, Bing Song, Tian Zhou

**Affiliations:** 1 Shenzhen Institute of Advanced Technology, Chinese Academy of Sciences, Shenzhen 518055, China; 2 College of Physics and Optoelectronic Engineering, Shenzhen University, Shenzhen 518061, China; 3 Institutes of Biomedical Sciences, Fudan University, Shanghai 200032, China

**Keywords:** platinum, iridium oxide, neural microelectrodes, nanostructure, biocompatibility

## Abstract

Neural interfaces provide a window for bio-signal modulation and recording with the assistance of neural microelectrodes. However, shrinking the size of electrodes results in high electrochemical impedance and low capacitance, thus limiting the stimulation/recording efficiency. In order to achieve critical stability and low power consumption, here, nanocone-shaped platinum (Pt) with an extensive surface area is proposed as an adhesive layer on a bare Pt substrate, followed by the deposition of a thin layer of iridium oxide (IrO_x_) to fabricate high-performance nanocone-array-based Pt-IrO_x_ neural microelectrodes (200 μm in diameter). A uniform nanocone-shaped Pt with significant roughness is created via controlling the ratio of NH_4_^+^ and Pt^4+^ ions in the electrolyte, which can be widely applicable for batch production on multichannel flexible microelectrode arrays (fMEAs) and various substrates with different dimensions. The Pt-IrO_x_ nanocomposite-coated microelectrode presents a significantly low impedance down to 0.72 ± 0.04 Ω cm^2^ at 1 kHz (reduction of ~92.95%). The cathodic charge storage capacity (CSC_c_) and charge injection capacity (CIC) reaches up to 52.44 ± 2.53 mC cm^−^^2^ and 4.39 ± 0.36 mC cm^−^^2^, respectively. Moreover, superior chronic stability and biocompatibility are also observed. The modified microelectrodes significantly enhance the adhesion of microglia, the major immune cells in the central nervous system. Therefore, such a coating strategy presents great potential for biomedical and other practical applications.

## 1. Introduction

Worldwide, there is a large unmet need for the detection, diagnosis, and treatment of neurological disorders. Our understanding of neuronal functions and dynamics is limited, largely due to our inability to monitor neural activities in both healthy and pathological disorders. Neural microelectrodes, acting as the key bridge of the neural interfaces to connect the inner tissues (biological) and external devices (technical), play a critical role in monitoring, diagnosing, and treating neurological diseases. They have been widely applied on various neural prostheses or interfaces, including retinal prosthesis for eliciting and reconstructing vision [1], cochlear implants for restoring hearing [2], deep brain stimulation for alleviating Parkinson’s disease and severe psychiatric disorders [3], peripheral nerve electrodes for acquiring and understanding chronic or acute disease states [4], and other invasive or implantable devices [5,6,7]. In recent years, neural microelectrodes have developed towards miniaturization and integration on the micro/nano scale in order to provide higher electrical stimulation efficiency for clinical practice. However, the large reduction in electrode size leads to a sharp increase in electrode impedance and inevitably the decrease of electrode capacitance, which magnifies the energy consumption and seriously limits its application [8,9,10]. Therefore, it is critical to effectively solve the contradiction between electrode size reduction and performance. 

At present, a surface modification approach is mainly utilized to improve the performance of neural microelectrodes without increasing their geometry size [8,11]. Various coating strategies, including noble metals, carbon materials, conductive polymers, and hydrogels, have been investigated to decrease the impedance [12]. Nevertheless, serious challenges still exist in practical use. Chronic stability and low adhesion are undesirable because long-term electrostimulation accelerates coating cracking and causes it to easily peel off [13,14]. Meanwhile, serious foreign body reactions exist during the implantation process [15]. Hence, it is urgent to develop high-performance nanomaterial-modified neural microelectrodes to be equipped with excellent properties such as low impedance, high capacitance, and high stimulation efficiency. Although flexible and soft materials are the prevailing development trends for neural electrodes, there are still many uncertain factors [16,17,18]. Traditional precious metal materials such as platinum (Pt) and iridium (Ir) still present great application value. As one of the most typical and demanding applications for implanted neural microelectrodes, retinal prostheses based on a multichannel flexible microelectrode array (fMEA) have been widely studied in the past few decades [1,19,20,21,22]. With rapid advancements in technology and engineering, the first commercial retinal prostheses system with 60-channel fMEA, called Argus II (Second Sight Medical Products Inc., Sylmar, CA, USA), was approved by Conformite Europeene (CE) in 2011 and the U.S. Food and Drug Administration (FDA) in 2013 [23,24]. This system employed Pt gray as a roughed layer to modify the fMEA, which improved the electrochemical performance to some extent. However, the impedance was still not low enough, and the capacitance was not high enough to meet the needs of the new generation of high-density fMEA for higher resolution [25,26]. 

Pt black, another form of nanostructure Pt, has an extensive surface area with a more porous morphology than Pt gray; nevertheless, it requires a toxic additive such as lead (Pb) to promote the growth of that crystal, strictly limiting its biomedical applications [27]. Roughing methods such as laser roughening [28] and electrochemically roughing [29] have been further proposed to improve the microelectrode surface. However, the former is rather complex, and the latter cannot enhance the electrochemical performance as expected. Because no electrochemical reaction occurs in the charge transfer process on the Pt surface (electric double layer capacitance, EDL), the capacitance is therefore limited [30]. Thus, an extensive contact area is urgently needed to expand its EDL capacitance. On the other hand, considering the superior faradaic pseudocapacitance of iridium oxide (IrO_x_), Lu et al. developed an electrodeposited iridium oxide film (EIROF) with a low impedance and enhanced charge injection ability [31]. Unsatisfactorily, the deposited layer was unstable and detached seriously. Conductive polymer such as poly(3,4-ethylenedionxythiophene) (PEDOT) is another promising material for neural interfaces, but the single solid coating has also been reported to delaminate facilely without any assistance measure due to its low stability [32,33]. 

In our previous work, we have already developed a nanocone-shaped Pt to improve the performance of electrodes. However, the morphology of the Pt structure has not been studied in detail, and the nanocrystal is disordered and heterogeneous. Consequently, the cathodic charge storage capacity (CSC_c_) and charge injection capacity (CIC) of the electrode are still not high enough for neural implants [26]. We also developed Pt nanoflowers with an extensive large surface area in order to improve the charge capacitance of electrochemical capacitors, the sensitivity of glucose detection, electrocatalytic activity towards oxygen evolution reaction (OER), and antibacterial properties [30,34]. However, the nanoflower structure has strict requirements on the electrodeposition process, because high voltage must be employed. Moreover, improper operation will easily cause the electrode coating to fall off and therefore damage the corresponding performance. In addition, the Pt nanoflower grows rapidly in all directions, and the deposition potential and duration time must be controlled precisely.

Considering that a well-controlled microstructure has a great influence on the high stimulation efficiency of neural interfaces, we have here focused on preparing uniform and consistent modified layers on the electrode surface with desirable impedance, CSC_c_ and CIC, stability, and biocompatibility. Because ammonium salts have been reported to promote the formation of nickel (Ni) nanocones with excellent consistency [35], in this study we combined the advantages of Pt and IrO_x_ to fabricate nanocone-array-based Pt-IrO_x_ neural microelectrodes. Firstly, two kinds of ammonium salts, including organic and inorganic, were chosen in order to obtain a nanocone-array-based Pt coating as the adhesive layer on bare substrate. Subsequently, we utilized a template-free and electrodeposition method to prepare the functional nanocone-array-based Pt neural microelectrode, which was achieved by controlling the proportion of NH_4_^+^ and Pt^4+^ ions in the electrolyte. An IrO_x_ layer with an extensive surface area was then deposited on the nanostructured Pt to achieve a high-performance neural interface. The morphology, electrochemistry, durability, and biocompatibility of the as-prepared nanomaterials were evaluated in detail before in vivo study. 

## 2. Materials and Methods

### 2.1. Microelectrode Fabrication

**Figure 1:**
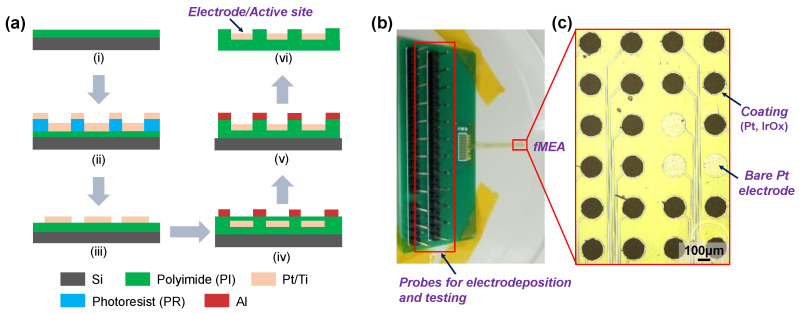
Flexible microelectrode array (fMEA). (**a**) Schematic of the microfabrication process, including spin-coating, photo-lithography, reactive ion etching and peeling off (i–vi). (**b**) Photographic image of fMEA (200 μm in diameter of electrode site), welded onto a printed circuit board (PCB). (**c**) Magnified view of coated (Pt, IrO_x_, or IrO_x_/Pt nanostructure) and uncoated (bare Pt) electrode sites.

Flexible polyimide (PI) was chosen as the packaging layer and insulating substrate to carry Pt microelectrodes (200 μm in diameter), which were fabricated as described in previous work [26,30]. As shown in Figure 1a, the process of creating 24-channel fMEAs began with spin-coating a thick PI layer (5 μm) as the substrate onto a silicon wafer, which was then cured at approximately 350 °C. A metal layer (Pt/Ti, ~100 nm) was then sputtered and patterned as the conductive layer of the fMEA using standard photo-lithography (EVG 610, St. Florian, Austria), followed by spin-coating another PI layer (~5 μm). The fMEA was etched to expose the electrode/active sites and the interconnection pads by oxygen reactive ion etching using Al as the hard mask. Finally, the fMEA was released from the silicon wafer for electrodeposition and testing in subsequent experiments. Figure 1b shows a photographic image of the fMEA; the interconnection pads are welded onto the customized printed circuit board (PCB, 24-channel) for electrodeposition and further characterization. The magnified view shown in Figure 1c represents the microelectrodes without modification (bare Pt) and with modified coatings (Pt, IrO_x_, or IrO_x_/Pt nanostructure). Ten fMEAs were fabricated simultaneously on the same silicon wafer, and three were selected randomly for testing. More than three microelectrodes were modified in each of the different electrodeposition processes in parallel. 

### 2.2. Electrodeposition of Pt and IrO_x_

The electrodeposition process was conducted on a three-electrode electrochemical workstation (Gamry Reference 600, Warminster, PA, USA), where the microelectrode (bare Pt and modified coating) was connected to the working electrode (WE) and an Ag/AgCl electrode, as well as a large-area Pt sheet. These were then employed as the reference electrode (RE) and counter electrode (CE). All the samples were carefully cleaned in acetone solution and electrochemically conditioned in 0.5 M H_2_SO_4_. 

All the Pt nanodendrites were formed in different Pt electrolytes; 10 mM PtCl_4_ and 20 mM (NH_4_)_2_PtCl_6_ were selected as the main sources of the Pt^4^^+^ ion, with 10 mM (NH_2_CH_2_)_2_·2HCl and 10 mM NH_4_Cl as the crystal promoters (purchased from Sigma, St. Louis, MO, USA). Different Pt morphologies could be formed by varying the ratio of components. The pH of the electrolytes was stabilized at ~7.4. The Pt layers were then electrodeposited at −0.6 V (vs. Ag/AgCl) for approximately 15 min by chronoamperometry.

The IrO_x_ layer was electrodeposited in an Ir electrolyte containing 4.25 mM IrCl_4_ (purchased from Sigma), with a pH of ~10.5. A slow scanning rate between 0.05 and 0.55 V was applied to obtain a stable IrO_x_ coating. 

All the samples were carefully rinsed with deionized water after each modification. 

### 2.3. Electrochemical Characterization

The electrochemical characteristics of different coated microelectrodes and bare Pt were assessed by a series of electrochemical methods, including electrochemical impedance spectroscopy (EIS), cyclic voltammetry (CV), and electrical pulse measurements. All measurements were performed in a 1× phosphate-buffered saline solution (PBS) at room temperature using the same three-electrode setup as described above. 

EIS measurements were conducted with a 10 mV amplitude signal over a frequency range of 100 kHz–1 Hz. The impedance at 1 kHz was used here to evaluate the resistance of the coated microelectrodes for neural application. CV sweeps were applied between −0.6 V and 0.8 V at a scan rate of 50 mV s^−1^. The cathodic charge storage capacity (CSC_c_) was defined as the area of the cathode region enclosed by one CV cycle. Prior to the tests, all the samples were soaked in PBS for at least 30 min to stabilize the electrode sites in solution. The CSC_c_ can be calculated by the following equation [26,34]:

(1) $${\mathrm{CSC}}_{c}=\frac{1}{\upsilon S}\int_{E_{c}}^{E_{a}}\left|i\right|dE$$

where *E* stands for the potential (V vs. Ag/AgCl), *E*_a_ and *E*_c_ are the corresponding anodic and cathodic potential limits, respectively, *i* is the current (A) responding to the potential, *S* represents the geometrical surface area of the microelectrode, and *υ* is the scanning rate of CV. 

In addition, the charge injection capacity (CIC) of the microelectrode was estimated by pulse-testing, which was determined by potential transient responses to a pulse current stimulation. It was realized with a neural stimulator (PENS0410, Greentek, Suzhou, China) to provide a symmetric biphasic current pulse (1 ms pulse width, 50 Hz) between a modified microelectrode and a Pt counter electrode in PBS. The potential excursion between the modified microelectrode and an Ag/AgCl reference electrode was monitored using a digital oscilloscope (TPS2012B, Tektronix, Suzhou, China). The maximum CIC was obtained by continuously increasing the pulse current density until the measured potential dropped at the boundary of “water window” (−0.6–0.8 V), after subtracting the ohmic voltage drop (“IR drop”) [34,36]. Consequently, CIC can be calculated with the following equation [26,34]:

(2) $$\mathrm{CIC}=\frac{I_{max}\cdot t}{S}$$

where *I_max_* is the maximum of charge injection current and *t* is the pulse width of the given signal.

### 2.4. Stability Evaluation

The mechanical stability of the coated microelectrodes was estimated in PBS by applying an intensive ultrasonic bath (500 W, 1 kHz) for 1 h. The corresponding variation rate of impedance and CSC_c_ of the microelectrode were then observed after it was stabilized in PBS for 30 min. In addition, a homemade bending/twist fatigue tester was also employed to evaluate the mechanical stability. The two ends of the microelectrode were clamped onto the equipment, followed by ±15 degrees of each twist cycle. The performance was then tested after 2000 cycles.

The electrochemical stability was then examined by applying long-time pulsing with continuous biphasic rectangular (cathodic-first) current pulses (500 µA, 200 µs) at 100 Hz for 0–30 days. After different pulsing times, the changes of CSC_c_ were characterized.

### 2.5. Morphology Characterization

Optical images of samples were taken with a microscope (Nikon Ni-U, Tokyo, Japan). The surface morphology and microstructure of the as-fabricated coating was observed by field emission scanning electron microscopy (FESEM, Zeiss SIGMA 300, Oberkochen, Germany). The root-mean-square (RMS) roughness of the samples was measured by atomic force microscopy (AFM, NanoManVS, Veeco, Plainview, TX, USA). 

### 2.6. Biocompatibility Study

Primary mouse microglia isolation and culture were performed as described below. Briefly, mouse brains were collected from P1~P3 neonatal C57BL/6J mice. After removing meninges and blood vessels, the mouse cortex was minced and dissociated by gentle mechanical disruption in 0.025% trypsin for 5 min. The cell suspension was then neutralized by 100% fetal bovine serum (FBS) and filtered through a 70 μm strainer. The culture was shaken at 180 rpm for 30 min on an orbital shaker. After centrifuging and removing supernatant, the culture was resuspended and cultured in Dulbecco’s Modified Eagle Medium (DMEM) with 10% FBS, 1% penicillin/streptomycin, and 20% LADMAC-conditioned media (produced from the LADMAC cell line CRL-2420). Following these steps, the resulting cells were purified microglia, as previously established, and they were comprehensively evaluated in our laboratory [37].

For the microglia adhesion assay, the purified microglia were cultured on the surface of different microelectrodes in DMEM medium for 24 h; the microglia that remained adhered on different surfaces were fixed with 4% paraformaldehyde (PFA). The fixed microglia were permeabilized with 0.1% Triton-X100 and blocked with 5% bovine serum albumin (BSA), followed by immunostaining with Iba1 (Wako, Cat#: 019-19741) antibody to label microglia and 4′,6-diamidino-2-phenylindole (DAPI). 

A Cell Counting Kit-8 (CCK8) viability assay was performed to assay the effect of microelectrode extracts on microglia viability. Microelectrodes were sterilized by soaking them in 75% ethanol overnight, followed by UV radiation for 3 h. The sterile microelectrodes were immersed in microglia culture medium for 48 h for extract collection. The collected extracts were co-cultured with microglia for 72 h, followed by CCK8 viability test (MEDChemExpress, Cat#: HY-K0301). The CCK8 kit uses a water-soluble tetrazolium salt that produces an orange formazan dye upon bio-reduction in the presence of an electron carrier. The formazan product is soluble in culture medium, and the amount of formazan produced is directly proportional to the number of living cells. The CCK8 assay was performed as instructed by the manufacturer.

## 3. Results and Discussion

### 3.1. Electrodeposition Process and Evaluation of Pt Nanocone

**Figure 2:**
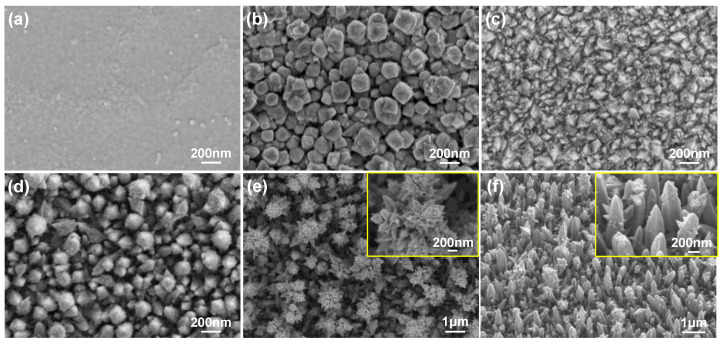
SEM images of coated microelectrodes with different deposition processes. (**a**) Bare Pt electrode. Morphology of nanostructured Pt deposited in electrolytes containing (**b**) PtCl_4_, (**c**) PtCl_4_ + (NH_2_CH_2_)_2_·2HCl, (**d**) PtCl_4_ + NH_4_Cl, (**e**) (NH_4_)_2_PtCl_6_ and (**f**) PtCl_4_ + (NH_4_)_2_PtCl_6_ respectively. The insets in (**e**,**f**) show the enlarged images of Pt nanocluster and nanocone respectively.

The micro-/nanostructure and morphology of microelectrode surfaces are quite important for the electrochemical performance of neural electrodes, and these can be realized by controlling the electrodeposition condition [34]. Figure 2 shows the representative morphologies and microstructures of different coated microelectrodes. Figure 2a represents the bare Pt microelectrode obtained using standard photo-lithography and reactive ion etching, with a flat surface and almost no microstructure. Figure 2b–f demonstrates different micrographs of the Pt nanodendrites achieved at the same constant potential of −0.6 V in different Pt electrolytes. It can be seen from Figure 2b that the coating deposited in the single PtCl_4_ electrolyte (10 mM) is covered by large and heterogeneous angular grains with sizes in the order of 50–200 nm. Such structure increases the contact area between the electrode and the working medium to some extent. It has been reported that ammonium salts can promote the directional growth of Ni nanocones [35]; we here have also chosen two types of crystal accelerator (organic and inorganic) in order to promote the directional growth of the Pt nanocrystal. When an amount of 10 mM (NH_2_CH_2_)_2_·2HCl was added onto the PtCl_4_ electrolyte, the microstructure did not improve in the expected direction (Figure 2c). While an appropriate amount of 10 mM NH_4_Cl was added, small nanocone-shaped crystals could be observed obviously attached to the surface, shown in Figure 2d. This may indicate that the inorganic ammonium salt has a better promotion effect on the crystal formation. Considering that (NH_4_)_2_PtCl_6_ contains NH_4_^+^ ion, we tested the effect of electrolyte containing 20 mM (NH_4_)_2_PtCl_6_ as the main Pt salt, and found that cluster-shaped Pt nanodendrites grew rapidly in all directions. Thus, the surface area could be increased by orders of magnitude without difficulty (Figure 2e). In order to control the Pt nanocone growing perpendicular to the substrate, we further controlled the amount of PtCl_4_ and (NH_4_)_2_PtCl_6_ in the same electrolyte, adjusting the ratio of n(NH_4_^+^) to n(Pt^4^^+^) to 4:5. Finally, a homogeneous and controllable nanocone-array-based Pt structure was achieved. A large nanocone-shaped structure can be easily formed, approximately 300 nm in width and 1 μm in height (Figure 2f). Such a nanocone array is directional electro-crystallization, and the aspect ratio of the nanocone structure can also be controlled. Therefore, both Pt nanocluster and nanocone structure are of particular interest due to their extensive surface area, which could further increase the charge capacity and reduce the impedance for improving neural stimulation/recording efficiency. 

The electrochemical performance is of great importance for neural microelectrodes. CV and EIS measurements were performed before and after modification in different Pt electrolytes in order to evaluate and confirm the proper electrolyte. 

**Figure 3:**
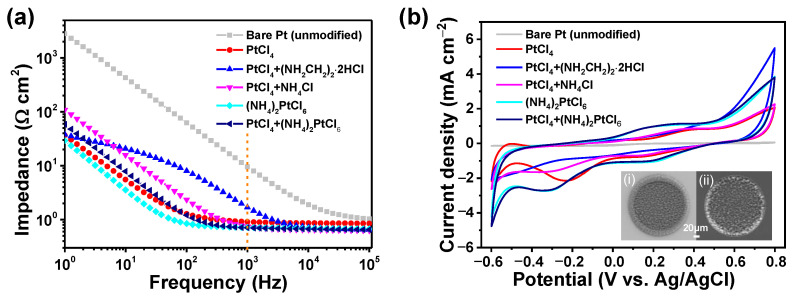
Electrochemical performance of microelectrodes before and after modification under different deposition process conditions. (**a**) EIS and (**b**) CV measurements on microelectrodes carrying different Pt nanodendrites in comparison with bare Pt (unmodified). The insets in (**b**) show the overall view of cluster−shaped and nanocone−shaped Pt deposited on single−channel electrodes, respectively.

The impedance at 1 kHz is one of the critical parameters for neural electrodes, because the typical electrophysiological signals largely occur at that frequency, which corresponds to the power consumption of neural stimulation and the noise of neural recording applications [26,36]. Figure 3a shows the electrochemical impedance in PBS of those modified microelectrodes in different Pt electrolytes at their open circuit potential. It can be seen that the impedance of bare Pt microelectrode at 1 kHz was approximately 9.63 Ω cm^2^, which decreased significantly following the nano-Pt modification. After deposition under the same potential and duration time, the impedance values were reduced to 0.91 Ω cm^2^ (PtCl_4_, reduction of 90.55%), 1.70 Ω cm^2^ (PtCl_4_ + (NH_2_CH_2_)_2_·2HCl, reduction of 82.35%), 0.75 Ω cm^2^ (PtCl_4_ + NH_4_Cl, reduction of 92.21%), 0.72 Ω cm^2^ ((NH_4_)_2_PtCl_6_, reduction of 92.52%), and 0.71 Ω cm^2^ (PtCl_4_ + (NH_4_)_2_PtCl_6_, reduction of 92.63%), respectively. It seems that the organic ammonium salt has no significant effect on the decrement of impedance after mixing with PtCl_4_, which is consistent with the SEM results. Meanwhile, other Pt electrolytes lead to a dramatic decrease of the impedance of almost more than 1 order of magnitude. In addition, more significant reduction can be observed for lower frequencies, e.g., 10 Hz and 100 Hz. This may be useful for neural electrodes in other operation modes, such as neuromuscular electrical stimulation (NMES), transcutaneous electrical nerve stimulation (TENS), and deep brain stimulation (DBS), etc. Lower impedance means higher signal-to-noise ratio (SNR) when recording weak neural signals, as well as lower energy consumption when stimulating the target tissue. More importantly, the safety of electrical stimulation can be improved with low impedance. 

In accordance with the low impedance, the CV measurements given in Figure 3b showed a distinct increase in CSC_c_ for the modified microelectrodes. The loop area of the unmodified Pt microelectrode was the smallest, with a minimum CSC_c_ of approximately 3.18 mC cm^−^^2^. Meanwhile, the CSC_c_ of nano-Pt-modified microelectrodes increased to ~25.48 mC cm^−^^2^ (PtCl_4_), ~25.02 mC cm^−^^2^ (PtCl_4_ + (NH_2_CH_2_)_2_·2HCl), and ~25.82 mC cm^−^^2^ (PtCl_4_ + NH_4_Cl), which were approximately 8.01, 7.87, and 8.12 times higher than that of bare Pt, respectively. The values of these three groups are relatively close. Nevertheless, the loop area of CV curves under (NH_4_)_2_PtCl_6_ and the mixture of PtCl_4_ + (NH_4_)_2_PtCl_6_ are similar and more extensively significant. The corresponding CSC_c_ are approximately 37.25 mC cm^−^^2^ and 38.32 mC cm^−^^2^, which are approximately 11.86 and 12.05 times higher than that of bare Pt, respectively. The insets in Figure 3b represent SEM images of microelectrodes deposited on (i) (NH_4_)_2_PtCl_6_ and (ii) the mixture of PtCl_4_ + (NH_4_)_2_PtCl_6_ electrolytes, respectively. It can be seen that the rapid growth in the (NH_4_)_2_PtCl_6_ electrolyte can easily result in the coating spilling over the edge of the microelectrode, due to the higher molarity of the NH_4_^+^ ion compared with the Pt^4^^+^ ion. This is undesirable in the implantation of neural electrodes. After properly controlling the ratio of both ions in the mixture of PtCl_4_ + (NH_4_)_2_PtCl_6_, the microelectrode surface was covered by a uniform and consistent nanocone-shaped array, completely deposited in the groove of the electrode site. This will facilitate the tissue contact and long-term service of the neural electrode during implantation. 

### 3.2. Optimization and Batch Production of Pt Nanocone

**Figure 4:**
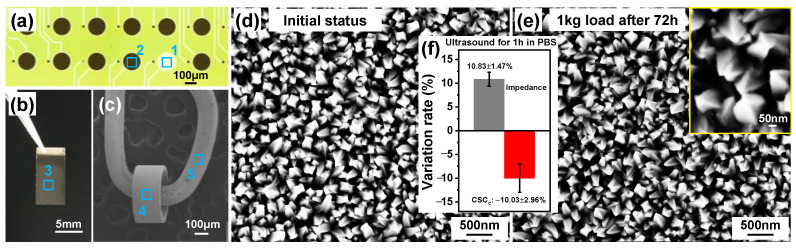
Batch production of Pt nanocones. Pt nanocone array electrodeposited on (**a**) electrophysiological fMEA, (**b**) platinized silicon wafers, and (**c**) Pt wire and cochlear implant. The numbers marked on panels (**a**–**c**) were chosen to observe the morphology, with point 1 as the unmodified electrode for reference. (**d**) The initial status and (**e**) after 1 kg load for 72 h of a Pt−nanocone−array−based platinized silicon wafer, the inset in (**e**) shows the enlarged image of Pt nanocone with excellent stability. (**f**) The variation rate of impedance and CSC_c_ after ultrasonic tests for 1 h in PBS.

In order to verify the universality of Pt nanocone for various geometries and substrates, we further fine-tuned the ratio of the solution and deposition conditions (n(NH_4_^+^)/n(Pt^4^^+^) = 4/5, −0.6 V, 10~15 min), and successfully completed the modification on electrophysiological fMEA, platinized silicon wafers, Pt wire, and cochlear implants. In Figure 4a, Point 1 is the bare Pt without modification, as a reference. Points (2–5), marked in Figure 4a–c, were chosen in order to observe the morphology. It was found that similar nanocone-array structures grew perpendicular to the surface and covered evenly on all samples with different channels and dimensions, including electrophysiological fMEA with different diameters (Figure 4a, 20 μm or 150 μm), platinized silicon wafers (Figure 4b), and a Pt wire and ring from a cochlear implant (Figure 4c). Subsequently, a 1 kg load was applied on a platinized silicon wafer for 72 h. The SEM result demonstrated almost no difference from the initial status (Figure 4d,e). The morphology illustrated that the coating consisted of billions of Pt nanocones with sizes of approximately 50–100 nm in width and 150–200 nm in height. Moreover, the AFM result also verified the homogeneity of nanocone structure. 

A high-intensity ultrasound test (500 W, 1 kHz) was performed in PBS for 1 h to further study the mechanical stability of coated microelectrodes, because an ultrasonic bath could easily remove the loosely attached substances on the surface. The corresponding variation rate is shown in Figure 4f. It can be seen that the impedance only increased by approximately 10.83%, and the CSC_c_ only reduced by approximately 10.03%, reflecting the strong adhesion between the Pt coating and the substrate. Assuming that the electrode is implanted in the eyeball, the eye will blink approximately 40,000 times if it works for 24 h a day (the eye blinks at most 30 times per minute). Meanwhile, 1 h of ultrasound at 1 kHz corresponds to 3.6 × 10^6^ times the amount of stimulation, which is equivalent to 90d of continuous usage. Hence, it presents excellent mechanical stability and shows superior potential for long-term applications. 

### 3.3. IrO_x_/Pt Nanocone for Better Neural Interface

Considering that low power consumption and chronic stability are critical requirements for neural implants, such as retinal prosthesis, we chose IrO_x_ as a second layer on the Pt nanocone array. Due to its high pseudo-capacitance characteristics, IrO_x_ can further improve the interface of neural microelectrodes equipped with lower impedance, higher CSC_c_, and higher CIC, as well as excellent chronic stability. 

**Figure 5:**
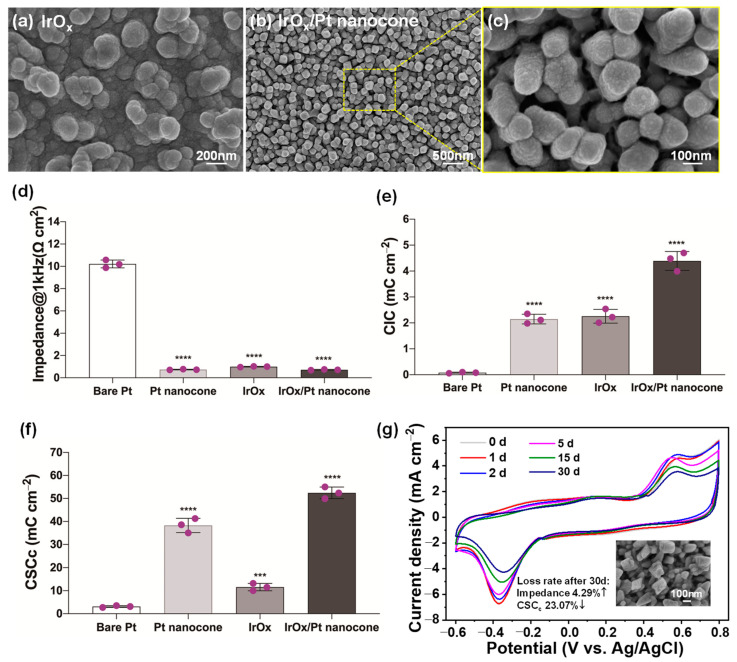
Morphology and electrochemical properties of different coated microelectrodes. SEM images of (**a**) IrO_x_ and (**b**,**c**) IrO_x_/Pt nanocone composites. (**d**–**f**) The comparison of impedance at 1 kHz, CSC_c_, and CIC of bare Pt, Pt nanocone, IrO_x_, and IrO_x_/Pt nanocone−coated microelectrodes. *** denotes *p* < 0.001, **** denotes *p* < 0.0001 by Student’s t test. (**g**) Chronic stability of IrO_x_/Pt nanocone−coated microelectrodes with different electrostimulation duration times at 100 Hz; the inset implies the stable structure of Pt nanocones.

Figure 5a shows an SEM image of IrO_x_ modified on a bare Pt microelectrode via CV cycles. The microstructure of the IrO_x_ coating was uniform and compact, with the size of nanoparticles (NPs) approximately 50–120 nm in diameter. Figure 5b demonstrates the morphology of an IrO_x_/Pt nanocone composite-coated microelectrode. IrO_x_ adhered tightly to the surface of the nanocone array, with good homogeneity and a rough contact area. The top view of the IrO_x_/Pt nanocone structure displayed in Figure 5c reveals a good adhesion between the nanocones and the IrO_x_ layer, which may be attributed to the nanocones having sharp angle (Figure 2f and Figure 4d). The Pt nanocone array structure cannot only provide a large surface area for accommodating more IrO_x_ NPs, but can also improve the adhesion and stability of these coating layers on bare Pt microelectrodes. 

The electrochemical properties of bare Pt microelectrodes, including impedance at 1 kHz (Figure 5d), CSC_c_ (Figure 5e), and CIC (Figure 5f), were compared to the herein proposed Pt nanocone, IrO_x_, and their composite coating in detail. The impedance of the IrO_x_ coating was reduced down to 0.99 ± 0.03 Ω cm^2^, which was more than one order of magnitude lower than that of bare Pt. The Pt nanocone-modified microelectrode exhibited a much lower impedance of 0.74 ± 0.03 Ω cm^2^ due to the extensive surface area of the nanocone array. Meanwhile, the introduction of IrO_x_ coating slightly reduced the impedance of Pt-IrO_x_ composites to 0.72 ± 0.04 Ω cm^2^, because the IrO_x_ coating was thin and did not obviously change the contact area of the nanocone. Such low impedance would help in reducing the energy consumption, and less pulse current would be needed to achieve the same stimulation intensity during practical applications.

A clear increment of the CSC_c_ of the coated microelectrodes can be observed in Figure 5e. The Pt nanocone increased CSC_c_ sharply up to 38.28 ± 3.12 mC cm^−^^2^, compared with that of bare Pt (3.12 ± 0.41 mC cm^−^^2^), while the addition of IrO_x_ further increased the CSC_c_ of Pt-IrO_x_ composites by approximately 1.37 times (52.44 ± 2.53 mC cm^−^^2^). Notably, a single IrO_x_ coating without particularly significant contact surface area could enlarge the corresponding CSC_c_ by approximately 3.7 times (11.55 ± 1.61 mC cm^−^^2^). This could be attributed to the high pseudo-capacitance of IrO_x_. Moreover, the IrO_x_/Pt nanocone-coated microelectrode exhibited the highest CIC (4.39 ± 0.36 mC cm^−^^2^), compared with that of bare Pt (0.08 ± 0.02 mC cm^−^^2^), Pt nanocone (2.15 ± 0.19 mC cm^−^^2^), and IrO_x_ (2.25 ± 0.26 mC cm^−^^2^). The nanocone-coated microelectrode here referred to the variant shown in Figure 2f in Section 3.1. Because the corresponding REDOX reactions occurred when the charge was injected through the reversible Faraday process, more charge could be accommodated between the interface of the electrode and tissue, at once combining the advantages of Pt and IrO_x_ [30,36]. The morphology and the results with identical deposition conditions are highly reproducible and controllable. In particular, these superior CSC and CIC properties would allow the neural electrode to store more energy and improve the stimulation/recording efficiency. A comparison of the main features with similar materials reported in the literature, including electrochemical impedance, CSC_c_, and CIC, are listed in Table 1. 

### 3.4. Chronic Stability Evaluation

The stability of neural electrodes is of great importance for chronic implantation applications, because the electrode surface will be subjected to hundreds of millions of electrical pulse stimulations. In addition, in the actual process, unavoidable friction occurs between the interface of the electrode and the tissue. In the above process, the mechanical stability of Pt nanocone has been verified. In this section we focused on the electrochemical stability of IrO_x_/Pt nanocone nanocomposite-coated microelectrodes, which was conducted in the same three-electrode electrochemical cell. It was carried out with an electronic stimulator to give the current pulse signal and a digital oscilloscope to collect the potential response. The CV measurements were performed after different pulse times, as shown in Figure 5g. 

From the data in Figure 5g, it is evident that a superior electrochemical stability of the IrO_x_/Pt nanocone nanocomposites can be observed during the first 5 days (4.32 × 10^7^ pulse cycles) of electrical stimulation, with less than 5% loss of CSC_c_ (~50 mC cm^−^^2^). Meanwhile, the CSC_c_ varied slightly to ~44.91 mC cm^−^^2^ (reduction of ~14.47%) when stimulation reached 15 d (1.30 × 10^8^ pulse cycles), which was still approximately 14.39 and 1.17 times higher than that of bare Pt and Pt nanocone-coated microelectrodes, respectively. Subsequently, we continued the stimulation process for up to one month (2.60 × 10^8^ pulse cycles), finding that the impedance of the nanocomposites only increased by approximately 4.29% and CSC_c_ reduced by approximately 23.07%. The inset of Figure 5g shows that the nanocone-array structure was almost unchanged, confirming its robust nature. Table 2 compares the CSC_c_ values of an IrO_x_/Pt nanocone-coated microelectrode with different electrostimulation duration times. Additionally, ultrasonic bath and bending/twist tests were also carried out; stable impedance and CSC levels can also be verified. Notably, there was no obvious degradation of the electrochemical performance after approximately 1 h of ultrasonic bath or 2000 torsional fatigue cycles. 

The stable and well-organized nanocone array is expected to promote excellent mechanical and electrochemical stability for long-term biomedical applications. Such structure is more conductive to uniform and close contact between the electrode and the nerve tissue/cells, which may further enhance the stimulation/recording efficiency. In future, we will continue to study the biotic stability in depth. Moreover, this simple strategy of nanocone-array-based structures can further be implemented on different conductive substrates with proper performance. 

### 3.5. Biocompatibility Study

Implantation of foreign devices causes tissue stress, and the inserted microelectrode can introduce potential cytotoxicity and foreign body response to brain cells. Microglia is the major resident immune cell type in the central nervous system, playing key roles in scavenging cell debris, maintaining neuronal networks, and orchestrating inflammatory response among others [43]. The effectiveness of the inserted microelectrode array could be limited by its biocompatibility to brain cells, including microglia. To assess the biocompatibility of the designed Pt/Ir nanocone-based microelectrode array in the central nervous system, we tested the effect of bare Pt microelectrodes and Pt nanocones (with or without IrO_x_ coating) on the adhesion and viability of in vitro cultured primary microglia that had been isolated from mouse brain. 

**Figure 6:**
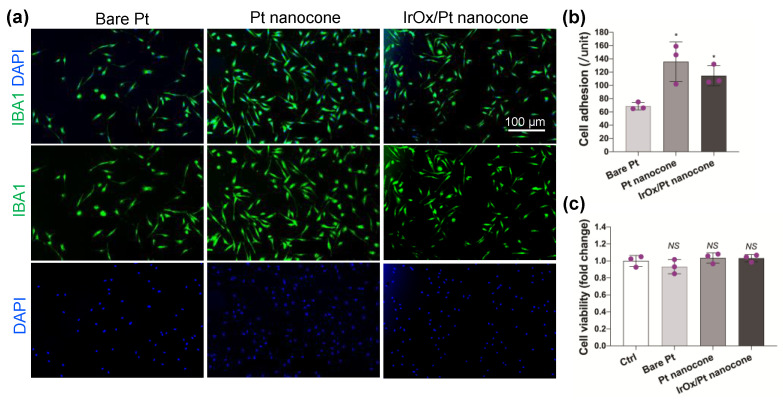
Biocompatibility evaluation of different microelectrodes on primary mouse microglia. Three microglia cultures were assayed for each experimental group. (**a**) Immunostaining of microglia-specific marker Iba1 (green) after culturing with extract from the indicated microelectrodes, and DAPI (blue)-stained nuclei. Scale bar is 100 µm. (**b**) Quantification of Iba1-positive microglia adhered to substrates following culture with different microelectrode extracts from (**a**). (**c**) Quantification of microglia viability following culture with the indicated microelectrode extracts using CCK8 viability assay. * denotes *p* < 0.05 by Student’s t test, NS denotes not significant.

For microglia adhesion, microglia were plated on the surface of different microelectrodes, and microglia adhesion was determined by measuring the microglia-specific marker Iba1. Our results clearly showed that the Pt nanocone-based microelectrode, with or without IrO_x_ coating, significantly improved microglia adhesion compared to the bare Pt microelectrode (Figure 6a,b). After culturing with different microelectrode extracts, we performed a CCK8 viability assay to determine the effects of different microelectrode extracts on microglia viability. It can be seen that bare Pt and nanocone Pt extracts showed no difference in microglia viability (Figure 6c). These results indicate that our designed nanocone Pt and Ir acts mainly on microglia adhesion rather than its viability, suggesting an interesting specificity on microglia biocompatibility.

## 4. Conclusions

In this study, we fabricated nanocone-array-based Pt-IrO_x_ neural microelectrodes, which were demonstrated to have high performance. NH_4_^+^ and Pt^4+^ ions were controlled to fabricate nanocone-shaped Pt, with a uniform and extensive surface area. It formed an adhesive layer on the bare Pt substrate, which was followed by the deposition of a thin layer of IrO_x_. The Pt-IrO_x_ nanocomposite-coated microelectrode presents a low impedance of 0.72 ± 0.04 Ω cm^2^ and an enhanced CSC_c_ and CIC of up to 52.44 ± 2.53 mC cm^−^^2^ and 4.39 ± 0.36 mC cm^−^^2^, respectively. The impedance of the nanocomposites only increases by approximately 4.29%, and CSC_c_ reduces by approximately 23.07% after a continued stimulation process of 2.60 × 10^8^ pulse cycles, showing superior chronic stability. In addition, excellent biocompatibility is also obtained. These nanocone-array-based Pt-IrO_x_ neural microelectrodes thus hold promise for alleviating neurological complications. In particular, the inspiring performance is attractive for various implantable/wearable devices, biosensing, energy storage, and other practical applications. Future studies should be directed at investigating the electrophysiological performance of these microelectrodes, both in vitro and in vivo. The capacity to measure neural activity is critical for the development and application of these microelectrodes. Microelectrodes embedded in Multi-Electrode Arrays (MEAs) have been routinely interfaced with isolated brain slices and implanted in the brains of freely moving animals. The MEA method enables the long-term recording of extracellular action potentials from a population of neurons at millisecond time scales and could be applied to the electrical stimulation of neuronal activity. 

## Figures and Tables

**Table 1:** Comparison of the main features with the similar materials reported in the literature.

Materials	Impedance at 1 kHz/Ω cm^2^	CSC_c_/ mC cm^−^^2^	CIC/ mC cm^−^^2^	Comments	References
IrO_x_/Pt black	32 (40-fold lower)	46.7	-	Pb additive, unsafe	[38]
Roughed Pt	Two orders of magnitude lower	44-fold increase	1	Low CIC	[29]
Pt nanograss	Two orders of magnitude lower at 100 Hz	40-fold increase	<0.5	Low CIC	[39]
Sputtered Pt	0.52 (9-fold lower)	11.4	-	Low CSC_c_	[40]
Deposited Pt-Ir	7.8-fold reduction	12.5 ± 0.75	3.58	Low CSC_c_	[41]
Activated IrO_x_	15.25 (reduction of 20%)	58.57 ± 0.96	-	High CSC_c_, but no significant reduction of impedance	[42]
IrO_x_/Pt gray	Reduction of 93.32%	22.29	~0.83	Low CIC, disordered structure	[26]
Nanocone-array-based Pt-IrO_x_	0.72 ± 0.04 (reduction of 92.95%)	52.44 ± 2.53	4.39 ± 0.36	Without template, low impedance, high CSC_c_ and CIC, superior stability and biocompatibility	This work

**Table 2:** Calculated CSC_c_ values of IrO_x_/Pt nanocone-coated microelectrode with different electrostimulation duration times.

Electrostimulation Time	0 Day	1 Day	2 Days	5 Days	15 Days	30 Days
CSC_c_/mC cm^−^^2^	52.44	52.05	50.96	50	44.91	40.34

## Data Availability

The data presented in this study are available on request from the corresponding author.

## References

[1] Yue L., Weiland J.D., Roska B., Humayun M.S. (2016). Retinal stimulation strategies to restore vision: Fundamentals and systems. Prog. Retin. Eye Res..

[2] Zeng F.-G., Rebscher S.J., Fu Q.-J., Chen H., Sun X., Yin L., Ping L., Feng H., Yang S., Gong S. (2015). Development and evaluation of the Nurotron 26-electrode cochlear implant system. Hear. Res..

[3] Sarica C., Iorio-Morin C., Aguirre-Padilla D.H., Najjar A., Paff M., Fomenko A., Yamamoto K., Zemmar A., Lipsman N., Ibrahim G.M. (2021). Implantable Pulse Generators for Deep Brain Stimulation: Challenges, Complications, and Strategies for Practicality and Longevity. Front. Hum. Neurosci..

[4] Cuttaz E.A., Chapman C.A.R., Syed O., Goding J.A., Green R.A. (2021). Stretchable, Fully Polymeric Electrode Arrays for Peripheral Nerve Stimulation. Adv. Sci..

[5] Wang J., He T., Lee C. (2019). Development of neural interfaces and energy harvesters towards self-powered implantable systems for healthcare monitoring and rehabilitation purposes. Nano Energy.

[6] Zhu M., Wang H., Li S., Liang X., Zhang M., Dai X., Zhang Y. (2021). Flexible Electrodes for In Vivo and In Vitro Electrophysiological Signal Recording. Adv. Healthc. Mater..

[7] Cho Y., Park S., Lee J., Yu K.J. (2021). Emerging Materials and Technologies with Applications in Flexible Neural Implants: A Comprehensive Review of Current Issues with Neural Devices. Adv. Mater..

[8] Zeng Q., Li X., Zhang S., Deng C., Wu T. (2022). Think big, see small—A review of nanomaterials for neural interfaces. Nano Sel..

[9] Woods G.A., Rommelfanger N.J., Hong G. (2020). Bioinspired Materials for In Vivo Bioelectronic Neural Interfaces. Matter.

[10] Zeng Q., Zhao S., Yang H., Zhang Y., Wu T. (2019). Micro/nano technologies for high-density retinal implant. Micromachines.

[11] Zheng X.S., Tan C., Castagnola E., Cui X.T. (2021). Electrode Materials for Chronic Electrical Microstimulation. Adv. Healthc. Mater..

[12] Chen N., Tian L., Patil A.C., Peng S., Yang I.H., Thakor N.V., Ramakrishna S. (2017). Neural interfaces engineered via micro- and nanostructured coatings. Nano Today.

[13] Oldroyd P., Malliaras G.G. (2022). Achieving long-term stability of thin-film electrodes for neurostimulation. Acta Biomater..

[14] Goding J.A., Gilmour A.D., Aregueta-Robles U.A., Hasan E.A., Green R.A. (2018). Living Bioelectronics: Strategies for Developing an Effective Long-Term Implant with Functional Neural Connections. Adv. Funct. Mater..

[15] Gori M., Vadalà G., Giannitelli S.M., Denaro V., Pino G.D. (2021). Biomedical and Tissue Engineering Strategies to Control Foreign Body Reaction to Invasive Neural Electrodes. Front. Bioeng. Biotechnol..

[16] Yuk H., Lu B., Zhao X. (2019). Hydrogel bioelectronics. Chem. Soc. Rev..

[17] Li H., Wang J., Fang Y. (2020). Bioinspired flexible electronics for seamless neural interfacing and chronic recording. Nanoscale Adv..

[18] Chen C., Sun X., Peng H. (2021). The Rise of Soft Neural Electronics. Giant.

[19] Ayton L.N., Blamey P.J., Guymer R.H., Luu C.D., Nayagam D.A., Sinclair N.C., Shivdasani M.N., Yeoh J., McCombe M.F., Briggs R.J. (2014). First-in-human trial of a novel suprachoroidal retinal prosthesis. PLoS ONE.

[20] Petoe M.A., Titchener S.A., Kolic M., Kentler W.G., Abbott C.J., Nayagam D.A., Baglin E.K., Kvansakul J., Barnes N., Walker J.G. (2021). A second-generation (44-channel) suprachoroidal retinal prosthesis: Interim clinical trial results. Transl. Vis. Sci. Technol..

[21] Titchener S.A., Nayagam D.A., Kvansakul J., Kolic M., Baglin E.K., Abbott C.J., McGuinness M.B., Ayton L.N., Luu C.D., Greenstein S. (2022). A Second-Generation (44-Channel) Suprachoroidal Retinal Prosthesis: Long-Term Observation of the Electrode–Tissue Interface. Transl. Vis. Sci. Technol..

[22] Arevalo J.F., Al Rashaed S., Alhamad T.A., Kahtani E.A., Al-Dhibi H.A., Mura M., Nowilaty S., Al-Zahrani Y.A., Kozak I., Al-Sulaiman S. (2021). Argus II retinal prosthesis for retinitis pigmentosa in the Middle East: The 2015 Pan-American Association of Ophthalmology Gradle Lecture. Int. J. Retin. Vitr..

[23] Rizzo S., Barale P.-O., Ayello-Scheer S., Devenyi R.G., Delyfer M.-N., Korobelnik J.-F., Rachitskaya A., Yuan A., Jayasundera K.T., Zacks D.N. (2020). Adverse Events of the Argus II Retinal Prosthesis: Incidence, Causes, and Best Practices for Managing and Preventing Conjunctival Erosion. Retina.

[24] Luo Y.H.-L., da Cruz L. (2016). The Argus^®^ II Retinal Prosthesis System. Prog. Retin. Eye Res..

[25] Zhou D.M. (2005). Platinum Electrode Surface Coating and Method for Manufacturing the Same.

[26] Zeng Q., Xia K., Sun B., Yin Y., Wu T., Humayun M.S. (2017). Electrodeposited iridium oxide on platinum nanocones for improving neural stimulation microelectrodes. Electrochim. Acta.

[27] Schuettler M., Doerge T., Wien S.L., Becker S., Stieglitz T. Cytotoxicity of Platinum Black. Proceedings of the 10th Annual Conference of the International Functional Electrical Stimulation Society: IFESS.

[28] Green R.A., Toor H., Dodds C., Lovell N.H. (2012). Variation in Performance of Platinum Electrodes with Size and Surface Roughness. Sens. Mater..

[29] Weremfo A., Carter P., Hibbert D.B., Zhao C. (2015). Investigating the Interfacial Properties of Electrochemically Roughened Platinum Electrodes for Neural Stimulation. Langmuir.

[30] Zeng Q., Huang Z., Cai G., Wu T. (2021). Platinum Nanocrystal Assisted by Low-Content Iridium for High-Performance Flexible Electrode: Applications on Neural Interface, Water Oxidation, and Anti-Microbial Contamination. Adv. Mater. Interfaces.

[31] Lu Y., Cai Z., Cao Y., Yang H., Duan Y.Y. (2008). Activated iridium oxide films fabricated by asymmetric pulses for electrical neural microstimulation and recording. Electrochem. Commun..

[32] Zeng Q., Wu T. (2022). Enhanced electrochemical performance of neural electrodes based on PEDOT:PSS hydrogel. J. Appl. Polym. Sci..

[33] Boehler C., Oberueber F., Schlabach S., Stieglitz T., Asplund M. (2017). Long-Term Stable Adhesion for Conducting Polymers in Biomedical Applications: IrOx and Nanostructured Platinum Solve the Chronic Challenge. ACS Appl. Mater. Interfaces.

[34] Zeng Q., Xia K., Zhang Y., Wu T. (2019). Well Controlled 3D Iridium Oxide/Platinum Nanocomposites with Greatly Enhanced Electrochemical Performances. Adv. Mater. Interfaces.

[35] Su Z., Yang C., Xie B., Lin Z., Zhang Z., Liu J., Li B., Kang F., Wong C.P. (2014). Scalable fabrication of MnO_2_ nanostructure deposited on free-standing Ni nanocone arrays for ultrathin, flexible, high-performance micro-supercapacitor. Energy Environ. Sci..

[36] Cogan S.F. (2008). Neural stimulation and recording electrodes. Annu. Rev. Biomed. Eng..

[37] Lian H., Roy E., Zheng H. (2016). Protocol for primary microglial culture preparation. Bio-Protocol.

[38] Yamagiwa S., Fujishiro A., Sawahata H., Numano R., Ishida M., Kawano T. (2015). Layer-by-layer assembled nanorough iridium-oxide/platinum-black for low-voltage microscale electrode neurostimulation. Sens. Actuators B Chem..

[39] Boehler C., Stieglitz T., Asplund M. (2015). Nanostructured platinum grass enables superior impedance reduction for neural microelectrodes. Biomaterials.

[40] Fan B., Rodriguez A.V., Vercosa D.G., Kemere C., Robinson J.T. (2020). Sputtered porous Pt for wafer-scale manufacture of low-impedance flexible microelectrodes. J. Neural Eng..

[41] Elyahoodayan S., Jiang W., Lee C.D., Shao X., Weiland G., Whalen J.J.I., Petrossians A., Song D. (2021). Stimulation and Recording of the Hippocampus Using the Same Pt-Ir Coated Microelectrodes. Front. Neurosci..

[42] Chen C., Ruan S., Bai X., Lin C., Xie C., Lee I.S. (2019). Patterned iridium oxide film as neural electrode interface: Biocompatibility and improved neurite outgrowth with electrical stimulation. Mater. Sci. Eng. C.

[43] Colonna M., Butovsky O. (2017). Microglia Function in the Central Nervous System during Health and Neurodegeneration. Annu. Rev. Immunol..

